# Continuation of GLP-1 Receptor Agonists During Post-Acute Skilled Nursing Facility Stays and 30-Day Outcomes

**DOI:** 10.1093/geroni/igaf122.4331

**Published:** 2025-12-31

**Authors:** Chan Mi Park, Eunji G Kim, Lan Luo, Xiecheng Chen, Dae Hyun Kim, Ellen McCarthy

**Affiliations:** Hebrew SeniorLife, Harvard Medical school, Boston, Massachusetts, United States; Marcus Institute for Aging Research, Boston, Massachusetts, United States; Marcus Institute for Aging Research, Boston, Massachusetts, United States; Hebrew Rehabilitation Center, Roslindale, Massachusetts, United States; Hebrew SeniorLife, Boston, Massachusetts, United States; Hebrew SeniorLife, Boston, Massachusetts, United States

## Abstract

In Medicare Part A– skilled-nursing facility (SNF) care—where facilities receive a predetermined, fixed amount per day —continuity of GLP-1 receptor agonists (GLP-1RAs) is unknown. Using the Long-Term Care Data Cooperative, a national provider-led EHR data linked to Medicare claims (>2,500 SNFs), we assembled a retrospective cohort of 7,037 older adults who received a GLP-1RA for ≥90 days before SNF admission. Exposure was percent days covered during the SNF stay: discontinuers (0%), partial continuers (>0–< 90%), and complete continuers (≥90%). Propensity scores with overlap weighting balanced demographics, frailty, primary diagnosis of hospitalization, healthcare utilization, facility ownership, glucose and kidney functions, cognition/ADLs, BMI, chronic conditions, and concomitant medications across three exposures (SMD < 0.10). After weighting, sample sizes were: discontinuers n = 612.6, partial n = 615.2, complete n = 612.6. Weighted baseline characteristics were similar: mean age 74.8 years; 38% male; race/ethnicity 79% non-Hispanic White; diabetes treatment profile ∼44% insulin, ∼33–34% metformin, and ∼37–38% other second-line therapy at baseline. The primary endpoint was a 30-day composite of death or cardiovascular hospitalization (heart failure, acute myocardial infarction, stroke); the safety endpoint was 30-day hospitalization for diabetic ketoacidosis or hypo/hyperglycemia. 30-day cumulative incidence for the composite was 10.66% (discontinuers) vs 8.26% (partial) vs 8.59% (complete). The hazard ratio for the composite was 0.77 (95% CI, 0.63–0.93) for partial and 0.80 (95% CI, 0.63–1.00) for complete compared to the discontinuers. Safety events were lower with continuation (1.19% vs 0.69% vs 0.63%). GLP-1RA continuation—partial or complete—was associated with lower 30-day mortality/CV risk than discontinuation, arguing against stopping during short SNF stays.

